# Left Ventricular Aneurysm Presenting as a Late Complication of Childhood Chemotherapy

**DOI:** 10.1155/2015/625451

**Published:** 2015-09-10

**Authors:** Braghadheeswar Thyagarajan, Lubna Bashir Munshi, Martin Miguel Amor

**Affiliations:** Department of Internal Medicine, Monmouth Medical Center, Long Branch, NJ 07740, USA

## Abstract

Cardiotoxicity is a well known adverse effect of chemotherapy. Multiple cardiac injuries have been reported including cardiomyopathy, pericarditis, myocarditis, angina, arrhythmias, and myocardial infarction. A left ventricular aneurysm due to chemotherapy is a rare and a dangerous complication which is particularly challenging in diagnosis requiring a high index of suspicion and periodic imaging. We present a case of a young Caucasian male with a past medical history of Acute Lymphocytic Leukemia status after chemotherapy during his childhood diagnosed with left ventricular aneurysm several years later.

## 1. Introduction

Cardiotoxicity is a well known adverse effect in children who receive chemotherapy for cancer [1]. Multiple cardiac injuries have been reported including cardiomyopathy, congestive heart failure, pericarditis, myocarditis, angina, arrhythmias, and myocardial infarction [2, 3]. The incidence of postmyocardial infarction left aneurysm varies from 3.5 to 5% in autopsy studies and 22 to 35% in the echocardiographic and radionuclide studies [4]. We present a case of a young Caucasian male with a past medical history of Acute Lymphocytic Leukemia status after chemotherapy during his childhood diagnosed with left ventricular aneurysm several years later.

## 2. Case Report

This is a case of a 27-year-old Caucasian male with past medical history significant for ALL who was brought into the ED by the EMS after an episode of cardiac arrest.

The patient's history starts when he was 5 years of age when he was diagnosed with Acute Lymphoblastic Leukemia. He underwent chemotherapy with vincristine, doxorubicin, and steroids at the time of diagnosis. At the end of two years, it was diagnosed that his ALL has progressed to involve the central nervous system and he was given chemotherapy for an additional two years which also included methotrexate. During this time the patient had complaints of constipation and after two weeks of no bowel movements, the patient was found straining in the toilet when he clenched his chest due to pain and fainted. He was taken to the hospital where he had a blood work and echocardiogram which were unremarkable. The patient recovered completely with no further similar episodes in his childhood. It is unclear whether the patient had an acute coronary syndrome or any arrythmias at that time. The patient eventually lost follow-up with his primary care doctor. There is no other past medical history and no positive family history for cardiac diseases. The patient also denied smoking, drinking of alcohol, and illicit drug use which was confirmed with the patient later.

**Figure 1:**
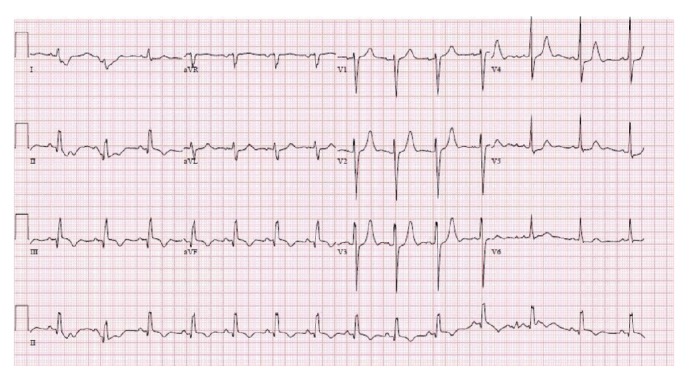
EKG showing normal sinus rhythm, Q waves in the inferior wall leads concerning old myocardial infarction.

On the day of admission, the patient was performing his usual duties in his workplace which included lifting of heavy objects. When the patient was walking after his work, he suddenly collapsed down on the floor. His friend who was present at the side witnessed this event. There was no breathing or palpable pulse. CPR was started and the paddles of the AED were connected which showed a shockable rhythm. The patient was shocked for four times and chest compression was given in between. When the patient was brought to the emergency department of the hospital he had an EKG (Figure 1) which showed normal sinus rhythm, Q waves in the inferior wall leads concerning old myocardial infarction versus a direct damage to the RCA territory myocardium due to chemotherapy effect. His vitals at the time of admission were blood pressure 86/47, heart rate 88, and respiratory rate 16 and he was afebrile. His labs were unremarkable with negative troponins. He was unresponsive at this time and his lungs and heart were clear to auscultation. He was intubated in the emergency department for airway protection and the patient was immediately admitted to the Intensive Care Unit.

**Figure 2:**
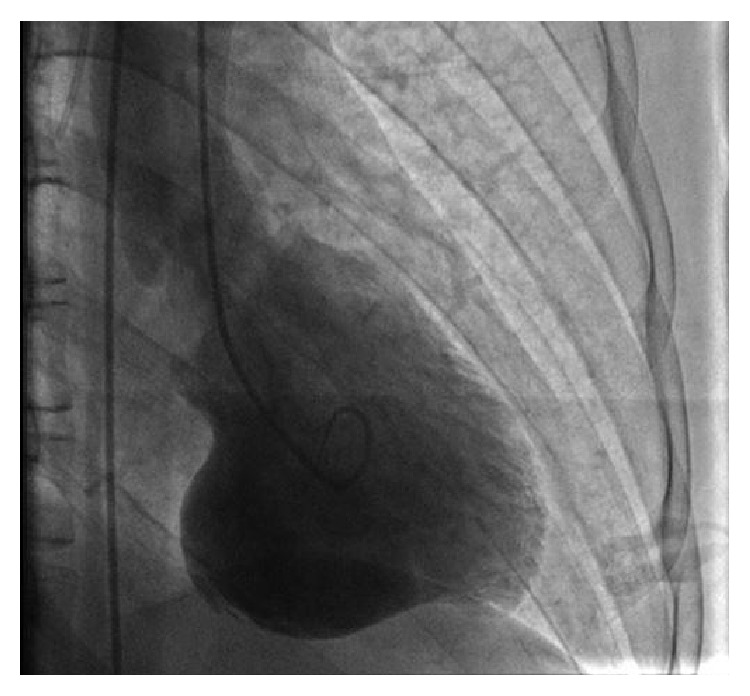
Left ventriculography with contrast showing left ventricular aneurysm.

**Figure 3:**
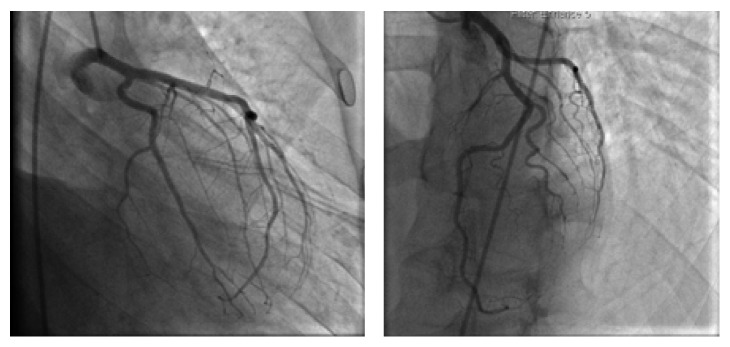
Normal LAD and LCX.

**Figure 4:**
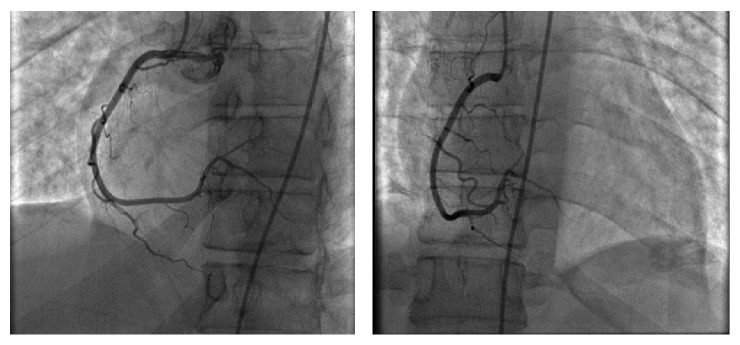
Normal RCA.

Eventually the patient got stabilized and was extubated. He had an echocardiogram which showed a LVEF of 36.9%, large basal and mid inferior aneurysm, akinesis of the inferior and inferolateral wall, moderate global hypokinesis of the left ventricle, and trace mitral regurgitation. He had a left and right cardiac catheterization along with left ventriculography (Figures 2, 3, and 4) which showed no evidence of disease in his left main, left anterior descending, and right coronary artery and left circumflex. His left ventriculography showed a normal sized ventricle, LVEF of 35 to 40%, and a large inferior wall aneurysm with the inferior base and midportion being akinetic. After the cardiac catheterization, the patient was transferred to a tertiary medical center for further care and he eventually received an Implantable Cardioverter Defibrillator with no surgical intervention and was managed medically with beta blocker and ACE inhibitor. Six months later after this event, the patient has been doing well with no further episodes of arrhythmias and has improved EF.

## 3. Discussion

ALL is the most common cancer diagnosed in children and represents approximately 25% of cancer diagnoses among children younger than 15 years. ALL occurs at an annual rate of 35 to 40 cases per 1 million people in the United States [5, 6]. Over the past 25 years, there has been a gradual increase in the incidence of ALL [3, 4, 7]. The 5-year survival rate for ALL has increased over the same time from 60% to approximately 90% for children younger than 15 years and from 28% to more than 75% for adolescents aged 15 to 19 years [8]. CNS involvement is approximately 3% in ALL [7].

Our patient received treatment with vincristine, doxorubicin, and steroids. And due to the involvement of CNS he had to receive treatment with intrathecal methotrexate for two years. Cardiotoxicity is a well known adverse effect in children who receive chemotherapy for cancer [1]. Doxorubicin has a direct cardiotoxic effect which is myocardial depression [9]. The incidence of congestive heart failure due to doxorubicin is dose dependent, ranging from 0.2% incidence of CHF in a dose of 150 mg/m^2^ to 8.7% in a dose of 600 mg/m^2^ [10]. The mechanism by which doxorubicin damages the cardiac myocyte is due to the formation of free oxygen radical and cardiac myocytes that are sensitive to these radicals are more prone to damage [11]. Another mechanism is by the effect of doxorubicin on mitochondrial performance which is by the interference with oxidative phosphorylation and inhibition of ATP synthesis. Free radicals released are thought to be responsible for many secondary effects such as lipid peroxidation, the oxidation of proteins and DNA, and depletion of glutathione and pyridine nucleotide reducing equivalents in the cell. These changes cause loss of mitochondrial integrity and function. And this causes an oncotic or necrotic cell death further leading to death of cardiac myocytes leading to cardiomyopathy [12]. It is shown that vincristine could protect the cardiac myocytes from the damage of oxidative stress from doxorubicin [13]. It is also shown that there is increased risk of myocardial infarction for about 25 years after treatment with doxorubicin [14]. High-dose methotrexate decreases levels of S-adenosylmethionine (a methyl donor) and increases sulfur-containing excitatory amino acids. These metabolic events have the potential to cause neurologic injury (e.g., demyelination) and overactivity (e.g., seizures). There is no information to support a role for these compounds in patients with cardiac toxicity. However, methotrexate inhibits the remethylation of homocysteine and causes acute and chronic elevations in homocysteine levels. Homocysteine also has important prothrombotic effects on the coagulation system due to its mechanism of getting rapidly autooxidized in plasma, and this process forms reactive oxygen species, such as superoxide and hydrogen peroxide. Chronic elevations of homocysteine levels can cause premature vascular disease, including stroke, myocardial infarction, and venous thromboembolism [15]. In our patient, who was treated with both doxorubicin and methotrexate during his childhood, the cardiotoxic effects could have been due to one drug or a combination of both.

Left ventricular aneurysm is defined as a distinct area of abnormal left ventricular diastolic contour with systolic dyskinesia or paradoxical bulging as visualised by ventriculography [16]. Left ventricular aneurysm usually results from myocardial infarction. Other rare etiologies of LVA include hypertrophic cardiomyopathy, Chagas' disease, sarcoidosis, congenital LVA and idiopathic dilated cardiomyopathy [17–20]. The incidence of left ventricular aneurysm is about 22% in anterior wall MI and the time period in which they usually occur is within 3 months after AMI [21] with only 3% of the LV aneurysm being inferior [22]. In an extensive review of medical literature we were not able to find any cases of left ventricular aneurysm as a direct consequence of doxorubicin or methotrexate. But there are articles about doxorubicin causing cardiomyopathy similar to the dilated type [23], isolated case reports where the cardiomyopathy was caused 17 years after the chemotherapy [24], and also articles about heart failure as a late complication due to doxorubicin [25]. As mentioned earlier in the discussion, doxorubicin can also lead to myocardial infarction due to damage of the myocytes; it is unclear in our patient whether he suffered myocardial infarction in childhood, when he had an episode of chest pain during straining episode of bowel movement. The patient at that time had no EKG changes and his echocardiogram was unremarkable. Unfortunately the patient never had a cardiac cath. during his childhood. The left ventricular aneurysm developed by the patient currently could have been a primary consequence because of the direct effect of chemotherapy on cardiac myocytes versus a secondary consequence because of a myocardial infarction or cardiomyopathy due to the toxicity of doxorubicin, irrespective of which our patient suffered a lethal cardiac arrhythmia due to his aneurysm.

A left ventricular aneurysm can cause a variety of complications such as arrythmias, angina pectoris, congestive heart failure, thromboembolism, and rupture. One-third of the patients develop arrhythmias and the arrhythmogenic focus is usually between the normal myocardium and the myocardium of the aneurysm [26]. Our patient suffered ventricular fibrillation which has a very high mortality. Contrast ventriculography is considered as gold standard for the diagnosis of left ventricular aneurysm; 2D echocardiogram also has high specificity and sensitivity [26]. Surgical management is only indicated for ventricular aneurysm associated with intractable ventricular tachyarrhythmias and/or pump failure [27]. In other scenarios, the management is with ICD to prevent VT/VF, medical management with beta blockers or calcium channel blocker along with anticoagulation in the presence of a thrombus [17]. Our patient, did not have intractable ventricular tachyarrhythmia or pump failure requiring emergent surgery. He eventually had an ICD placed and was medically managed as outpatient. Six months after this event, the patient did not have further episodes of arrhythmias and his ejection fraction also improved.

## 4. Conclusion

Chemotherapy during childhood can cause lethal cardiac complications even after decades of completing the treatment. Periodic monitoring with imaging studies such as 2D echocardiogram should be done in these patients to assess the ventricular function and formation of aneurysm or detect dilated cardiomyopathy so that appropriate preventive measures can be taken to reduced mortality. The need for the same should be stressed with the patients as well as their parents.
